# Mortality in persons with undetected and diagnosed hypertension, type 2 diabetes, and hypothyroidism, compared with persons without corresponding disease - a prospective cohort study; The HUNT Study, Norway

**DOI:** 10.1186/s12875-017-0672-7

**Published:** 2017-12-07

**Authors:** Pål Jørgensen, Arnulf Langhammer, Steinar Krokstad, Siri Forsmo

**Affiliations:** 1 Department of Public Health and Nursing, NTNU, Norwegian University of Science and Technology, Postbox 8905, 7491 Trondheim, Norway; 2 HUNT Research Centre, Department of Public Health and Nursing, NTNU, Norwegian University of Science and Technology, Levanger, Norway; 3 Levanger Hospital, Nord-Trøndelag Hospital Trust, 7600 Levanger, Norway

**Keywords:** Chronic disease, Diabetes, Hypertension, Thyroid disorders, Primary care, Public health

## Abstract

**Background:**

Suggested strategies in reducing the impact of non-communicable diseases (NCD) are early diagnosing and screening. We have limited proof of benefit of population screening for NCD. Increased mortality in persons with diagnosed NCD has been shown for decades. However, mortality in *undetected* NCD has barely been studied. This paper explores whether all-cause mortality differed between persons with diagnosed hypothyroidism, type 2 diabetes (T2DM), and hypertension, compared with persons with undetected-, and with persons without the corresponding disease.

**Methods:**

A prospective cohort study of the general population in Nord-Trøndelag, Norway. Persons ≥20 years at baseline 1995–97 were followed until death or June 15, 2016. Cox proportional hazards models were used to compute age and multiple adjusted hazard ratios (HR) with 95% confidence intervals (CI) for the association between disease status and all-cause mortality. The number of participants in the hypothyroidism study was 31,960, in the T2DM study 37,957, and in the hypertension study 63,371.

**Results:**

Mortality was increased in persons with diagnosed type 2 diabetes and hypertension, compared to persons without corresponding disease; HR 1.69 (95% CI 1.55–1.84) and HR 1.23 (95% CI 1.09–1.39), respectively. Among persons with undetected T2DM, the HR was 1.21 (95% CI 1.08–1.37), whilst among undetected hypothyroidism and hypertension, mortality was not increased compared with persons without the diseases. Further, the association with mortality was stronger in persons with long duration of T2DM (HR 1.96 (95% CI 1.57–2.44)) and hypertension (HR 1.32 (95% CI 1.17–1.49)), compared with persons with short duration (HR 1.29 (1.09–1.53) and HR 1.16 (1.03-1-30) respectively).

**Conclusions:**

Mortality was increased in persons with diagnosed T2DM and hypertension, and in undetected T2DM, compared with persons without the diseases. The strength of the association with mortality in undetected T2DM was however lower compared with persons with diagnosed T2DM, and mortality was not increased in persons with undetected hypothyroidism and hypertension, compared with persons without the diseases. Thus, future research needs to test more thoroughly if early diagnosing of these diseases, such as general population screening, is beneficial for health.

## Background

Hypothyroidism, type 2 diabetes mellitus (T2DM), and hypertension generally cause few symptoms, remains asymptomatic for a long time, and may not be detected without some sort of screening. The non-communicable diseases (NCDs) T2DM and hypertension are among the leading causes of death in developed countries [1, 2]. As NCDs are modifiable risk factors for premature death, screening programs are regularly suggested by “task forces” [3, 4]. Screening seems intuitively positive as mortality is increased in persons with the diagnoses [5, 6].

Except for in mild hypertension, clinical trials have demonstrated benefit over harm of antihypertensive drug treatment [7–9]. However, outside clinical trials, only half of the patients were found to have their blood pressure controlled (<140/90 mmHg) when treated for hypertension [10].

Although several antidiabetic drugs are shown to reduce hyperglycaemia in T2DM, there is a paucity of high-quality studies showing benefit of medication on clinical important/long-term outcomes such as macrovascular complications and mortality [11]. Outside clinical trials, an American study from 2014 found that only 35% of T2DM patients reached the treatment goal (hemoglobin A1c (HbA1c) <7%), despite life-style and medical interventions [12]. Similar results have been reported from Norway [13]. Convincing evidence that population screening for T2DM and hypertension reduces mortality has not yet been found [4, 14, 15]. Krogsbøll et al. neither found effect on mortality nor morbidity, of general health checks in adults [16]. Although the evidence clearly has been conflicting, [15, 17, 18] some epidemiological studies have shown increased mortality among persons with *undiagnosed* hypothyroidism, T2DM, and hypertension [19–22].

Besides neonatal screening of hypothyroidism, no official screening programs on hypothyroidism, T2DM or hypertension are implemented in Norway. The numbers diagnosed in preclinical stages of disease however, seems to be increasing. This is in line with recommendations from patient support organizations, authoritative medical specialists, and commercial interests.

We have previously shown that persons diagnosed with hypothyroidism, T2DM, and hypertension more often report poor self-rated health (SRH) compared to persons without, and to persons with undetected corresponding disease [23, 24]. Since the eighties, studies have consistently shown an association between poor SRH and increased mortality [25–27]. However, the underlying mechanisms are far from clarified.

In our population, mortality among persons with undetected hypothyroidism, T2DM, and hypertension has not been investigated (see Table 1 for definition of undetected disease). Even if the design of the present study does not allow causal inference, the results would be relevant as part of evaluation of benefit of screening or early case finding of these conditions in adults. We aimed to compare all-cause mortality between persons with diagnosed and persons with undetected hypothyroidism, T2DM, and hypertension in a general population, compared with persons without the corresponding disease.

**Table 1:** Classification of baseline disease status. HUNT2, 1995–97

Disease status	Self-reported disease status	Measurement
Hypothyroidism	Ever had any thyroid disease?	
No	No	TSH 0.2 – 4.5 mU/L and FT_4_ 8.0 – 20.0 pmol/L
Undetected^a^	No	TSH >4.5 mU/L and FT_4_ < 8 pmol/L
Diagnosed	Yes	–
Type 2 diabetes mellitus	Ever had diabetes mellitus?	Serum glucose ≥2 h after last meal
No	No	≤7.0 mmol/L
Undetected^a^	No	>7.0 mmol/L
Diagnosed	Yes	–
Hypertension	BP follow-up necessary?^b^	Arterial BP
No	No	<140 mmHg systolic and <90 mmHg diastolic
Undetected^a^	No	≥140 mmHg systolic and/or ≥90 mmHg diastolic
Diagnosed	Yes	–

*TSH* thyroid stimulating hormone, *FT*_*4*_ free T_4_, *BP* blood pressure

^a^In participants with undetected disease, the disease was not reported by the participant and we assume it was unknown also for their physician. The HUNT Study data however, indicated the disease

^b^BP follow-up necessary include start/continue medication or recommended BP follow-up answer alternatives. Participants with missing data were excluded

## Methods

### Study population

**Fig. 1:**
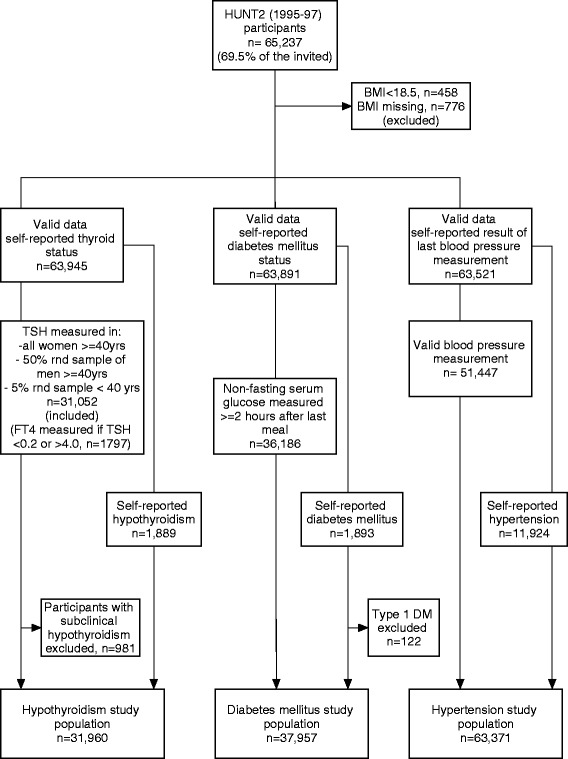
Inclusion and exclusion criteria of the study population. HUNT2, 1995–97; BMI, body mass index; TSH, thyroid stimulating hormone; FT4, free T4; DM, diabetes mellitus; rnd., random. Subclinical hypothyroidism were defined as no self-reported thyroid disease, TSH > 4.5 mU/L and FT4 8.0 – 20.0 pmol/L. Type 1 DM has been defined by HUNT Databank as starting insulin treatment within 1 year of diagnosis if, in addition, either anti-GAD/anti-IA-2 positive or antibody negative and fasting C-peptide levels <150 pmol/l

Between August 1995 and June 1997, all inhabitants in the Nord-Trøndelag County, Central Norway, aged above 19 years, were invited to the second wave of the Nord-Trøndelag Health Study (HUNT2). The population is fairly representative for the general Norwegian population regarding demography, socio-economic factors, morbidity and mortality, however in the county there are no large cities [28]. Altogether 65,237 participants (69.5% of the invited) completed health-related questionnaires, inter alia on hypothyroidism, DM, and hypertension. Thyroid stimulating hormone (TSH) was measured in a subset of participants (Fig. 1), whereas blood pressure (BP), non-fasting serum glucose, height and weight were measured in all participants. We excluded underweight persons (BMI < 18.5 kg/m^2^) from the analyses (*n* = 458) due to increased risk for various end-stage conditions in this group.

Between October 2006 and June 2008, HUNT3 was carried out with a total of 50,807 participants (54.1% of the invited), answering a similar questionnaire. Now, all participants had TSH measured, otherwise measurements were similar as in HUNT2.

### Hypothyroidism

All participants answered questions on history of thyroid disease (hypo- and hyperthyroidism, goitre, and other thyroid disease). TSH and free T_4_ (FT_4_) were measured in a subset of participants (Fig. 1), and were analysed at the Hormone Laboratory, Aker University Hospital, Norway. The laboratory reference range for TSH was 0.2–4.5 mU/L and for FT_4_ 8.0–20.0 pmol/L. If TSH was <0.2 mU/L or >4.0 mU/L, serum FT_4_ was also analysed. Categorization of hypothyroidism status is shown in Table 1.

### Type 2 diabetes mellitus

All participants answered question on history of DM, and had non-fasting serum glucose measured between ten a.m. and six p.m. The serum analysis took place at Levanger Hospital, Norway. In clinical settings, fasting serum glucose is used as a diagnostic measure. However, non-fasting serum glucose above a cut-off at 5.6 mmol/L showed reasonable sensitivity (68–74%) and specificity (66–77%) in predicting T2DM [29]. Further, rather small differences in serum glucose levels have been found between fasting and non-fasting individuals without known DM [30]. In our study population, the median non-fasting serum glucose value was 5.2 mmol/L with a 95-percentile value of 7.5 mmol/L (*n* = 63,882). To enhance specificity of the DM classification, we used the 95 percentile among participants having fasted 2 h or more in the T2DM study part as cut-off, i.e. 7.0 mmol/L. Participants reporting DM that had started insulin treatment within 1 year of diagnosis and, in addition, had either anti-GAD/anti-IA-2 positive or antibody negative and fasting C-peptide levels <150 pmol/l (type 1 DM), were excluded. See Fig. 1 and Table 1 for inclusion and categorization criteria of T2DM status.

According to HUNT2 protocol, participants with glucose ≥11.1 mmol/L were recommended to visit their GP. Sixty-two out of 648 participants in the undetected T2DM category, included in the full model Cox PH analyses, had such glucose reading, and 35 of them died during follow-up. Only two of the participants recommended to visit their GP did not report DM in HUNT3, suggesting a screening detection-effect of the survey.

### Hypertension

All participants were asked about the doctor’s advice after the latest BP measurement prior to participation in HUNT2 and standardized BP measurements were performed at screening stations; mean systolic (s) and mean diastolic (d) arterial BP of measurement two and three were used as BP-measures [28]. Cut-off values defining hypertension were made according to the European society of hypertension’s definitions; sBP ≥ 140 mmHg and/or dBP ≥ 90 mmHg. Hypertension inclusion and categorization criteria are shown in Fig. 1 and Table 1.

According to HUNT2 protocol, participants with dBP ≥125 mmHg were recommended to visit their GP. None of the participants in the undetected hypertension group had dBP > 125.

### Covariables

In addition to administrative data on age and sex, data on body mass index (BMI), smoking habits, educational status, limiting long-term illness or injury, physical activity, serum cholesterol and serum creatinine were collected from the questionnaire and clinical examination at baseline. Age was included as a continuous variable. BMI (kg/m^2^) was calculated of measured height and weight and categorized according to The WHO’s definition; normal weight (18.5-24.9 kg/m^2^), overweight (25.0-29.9 kg/m^2^) and obese ≥30.0 kg/m^2^). Smoking status was categorized into non-daily smokers (never- and occasional smokers), former daily smokers, and current daily smokers. We categorized educational level according to years of education; (≤12 or >12 years). Participants reporting limiting long-term illness or injury (Question: “Do you suffer from any long-term illness or injury (at least one year) of a physical or psychological nature that impairs your functioning in your everyday life?”) were categorized as “prevalent long-term illness”, otherwise “no long-term illness”. Physical activity was categorized 1; hard activity 3 h or more per week, and/or walk or lift a lot at work, or 2; hard activity less than 3 h per week, and less active work. Serum cholesterol (mmol/L) and serum creatinine (μmol/L) was included as continuous variables. The covariates were selected on the basis of a priori evaluation, supported by Directed Acyclic Graphs considerations [31].

### Outcome

Participants were followed from the date of attendance in HUNT2 to the date of death or end of follow-up; June 15, 2016. Data on participant’s vital status (alive, emigrated, dead), was given by the National Registry through HUNT databank [32]. The outcome variable was all-cause mortality.

### Statistical analyses

Descriptive analyses of baseline characteristics were performed stratified on status of hypothyroidism, T2DM, and hypertension. Cox proportional hazards (PH) models were used to compute age and multiple adjusted hazard ratios (HR) with 95% confidence intervals (CI) for the association between disease status and all-cause mortality. The first models included age only as a covariate; thereafter the remaining covariates were additionally included. All analyses of hypothyroidism were gender stratified (rather than adjusted) owing to the uneven distribution of TSH and FT_4_ measurements between women and men.

Statistically significant interactions (*p* < 0.1) between disease statuses and covariates were included in the cox PH models.

Even though the log-log plots showed no violation of the PH assumption, there was a statistically significant interaction between observation-time and hypertension status in HUNT2 (*p* = 0.01) in the time dependant cox model; hence the interaction term was included.

### Supplementary analyses

Participants reporting “recommended follow-up examination but not to start medicine” on the hypertension question might be misclassified by our original classification “diagnosed hypertension”. Hence, in a sensitivity analysis we reclassified them into “normotensive” if BP <140/90, or “undetected hypertension” if BP ≥140/90.

Secondly, to investigate the effect of disease duration, we estimated HR for mortality by categories of disease-statuses in those participating at both HUNT2 and HUNT3. Persons without the diagnoses at both surveys were chosen as reference category. The remainders were (a) participants with undetected disease at baseline and/or follow-up, (b) that had become diagnosed during follow-up, or (c) that were diagnosed at baseline and still reported the diagnosis at HUNT3.

All analyses were conducted using IBM SPSS Statistics version 22.

## Results

### Baseline characteristics

Age was higher among participants with diagnosed T2DM or hypertension, compared to undetected and persons without the diseases (Table 2). For hypothyroidism, age was highest in the undetected group. More men than women had undetected T2DM (3.3% versus 2.2%; *p* < 0.05) and undetected hypertension (34% versus 24%; *p* < 0.05). For undetected hypothyroidism the figures were opposite (0.2% in men and 0.7% in women; *p* < 0.05). BMI was higher in undetected and diagnosed persons, than in persons without disease, except in men with undetected hypothyroidism. Smoking was most common in persons without disease, and the proportion that had quitted smoking was highest among persons diagnosed with T2DM or hypertension. Persons without T2DM or hypertension were higher educated than undetected and diagnosed persons, but for hypothyroidism diagnosed persons were more educated. Overall, long term illness was less frequently reported among persons without these diseases than among persons with undetected or diagnosed disease. However, women with undetected hypothyroidism reported long term illness less frequently than persons without the disease. Persons without T2DM and hypertension were more physically active, and they had lower serum cholesterol and creatinine, compared with undiagnosed and diagnosed disease.

**Table 2:** Baseline characteristics of the study population. HUNT2 1995–97, Norway

Disease status	*N*	Age, years mean (SD)	Women,%	BMI, kg/m^2^ mean (SD)	Smoker, %		Higher education, %	Long-term illness, %	Daily physical activity, %	Serum cholesterol, mmol/L (SD)	Serum creatinine, μmol/L (SD)
					Daily	Ex					
Hypothyroidism women											
No	19,387	58.3 (13.5)	–	26.8 (4.5)	28.3	23.1	15.3	43.1	42.9	6.3 (1.3)	83 (13)
Undetected	151	61.4 (13.0)	–	28.0 (4.8)	17.2	29.7	13.7	38.7	45.7	6.8 (1.5)	86 (11)
Diagnosed	1631	57.1 (15.1)	–	27.9 (5.1)	24.0	27.7	17.8	54.0	41.4	6.3 (1.3)	84 (14)
Hypothyroidism men											
No	10,510	57.6 (13.4)	–	26.8 (3.5)	29.4	40.5	18.7	42.4	56.0	6.1 (1.1)	96 (17)
Undetected	23	60.7 (13.8)	–	26.7 (2.4)	17.4	47.8	4.4	57.1	61.1	6.2 (1.0)	111 (28)
Diagnosed	258	59.6 (15.9)	–	27.7 (4.0)	23.1	47.8	19.1	54.7	52.0	5.9 (1.2)	100 (16)
Type 2 diabetes mellitus											
No	35,164	50.0 (17.1)	53.0	26.4 (4.0)	30.1	27.0	19.4	35.2	55.3	5.9 (1.3)	88 (15)
Undetected	1022	64.4 (14.4)	42.5	28.4 (4.8)	24.1	36.4	9.0	54.9	40.4	6.4 (1.2)	93 (17)
Diagnosed	1771	67.0 (13.4)	51.8	29.1 (4.8)	17.6	36.7	9.6	66.9	34.0	6.2 (1.3)	94 (26)
Hypertension											
No	33,368	42.3 (14.1)	57.9	25.4 (3.6)	32.9	23.8	25.6	26.4	60.9	5.5 (1.2)	85 (13)
Undetected	18,079	56.6 (16.4)	44.2	27.1 (3.9)	26.9	30.0	14.3	40.1	52.2	6.3 (1.2)	90 (14)
Diagnosed	11,924	61.4 (14.9)	52.2	28.3 (4.4)	21.3	34.3	12.0	52.8	41.5	6.3 (1.3)	93 (21)
Missing		None	None	None	2.1%		5.2%	4.1%	14.2%	0.2%	0.2%

Mean with standard deviation (SD) for age in years, body mass index (BMI) in kilograms per meter^2^, serum cholesterol in mmol/L, and serum creatinine in μmol/L. Proportion in per cent of women, daily and ex-smokers, participants with higher education (>12 years), long-term limiting illness or injury, and daily physical activity. Participants with missing data were excluded

### Covariables

Overall, all-cause mortality increased by age, daily smoking, long-term illness, male sex, increasing serum creatinine and low education in all adjusted cox PH models (data not shown). Compared to those with normal weight, those with overweight were less likely to die, whereas obese persons had increased mortality. Less physically active persons had increased mortality compared with more physically active. Serum cholesterol was not associated with mortality in any analyses.

### Hypothyroidism

Neither persons reporting hypothyroidism, nor persons with undetected hypothyroidism at HUNT2 had increased mortality, compared to persons without the diseases (Table 3). Neither in the fully adjusted analyses of persons attending both HUNT2 and HUNT3 there were increased mortality in any of the hypothyroidism statuses, compared to health persons (Table 4). Adjusted for age only, women with diagnosed hypothyroidism in both surveys had increased mortality (HR 1.30 (1.04–1.61). Particularly in men, very few cases gave too low statistical power to reveal any associations.

**Table 3:** Associations between disease status at baseline and all-cause mortality. The HUNT Study, Norway

			HR (95% CI)^a^	
Disease statuses	Person-years^b^	Deaths^b^	Model 1^c^	Model 2^c^
Hypothyroidism women				
No	243,950	3029	1.00	1.00
Undetected	1598	21	0.94 (0.72–1.21)	0.92 (0.60–1.42)
Diagnosed	19,753	256	1.06 (0.97–1.16)	1.05 (0.92–1.19)
Hypothyroidism men				
No	141,050	2518	1.00	1.00
Undetected	291	5	0.94 (0.49–1.81)	0.63 (0.26–1.51)
Diagnosed	2806	73	1.04 (0.88–1.25)	1.06 (0.84–1.33)
Type 2 diabetes mellitus				
No	504,884	4971	1.00	1.00
Undetected	9651	299	1.28 (1.18–1.39)	1.21 (1.08–1.37)
Diagnosed	12,968	602	1.70 (1.60–1.80)	1.69 (1.55–1.84)
Hypertension				
No	556,423	2375	1.00	1.00
Undetected	231,569	3523	0.88 (0.81–0.97)	0.93 (0.82–1.05)
Diagnosed	128,959	3121	1.17 (1.07–1.28)	1.23 (1.09–1.39)

^a^HR, hazard ratio; CI, confidence interval

^b^Participants with missing data in fully adjusted analyses were excluded

^c^Model 1; adjusted for age. Model 2; Model 1 + sex, body mass index, smoking status, educational level, long term limiting illness, physical activity, serum cholesterol and creatinine

**Table 4:** Associations between disease status and all-cause mortality in participants attending both HUNT2 (1995–97) and HUNT3 (2006–08)

			HR (95% CI)	
Disease statuses	Person-years^a^	Deaths^a^	Model 1^b^	Model 2^b^
Hypothyroidism women				
No	145,398	591	1.00	1.00
Undetected	2219	6	0.94 (0.57–1.54)	0.55 (0.25–1.23)
Diagnosed during follow-up	16,201	50	1.09 (0.87–1.36)	0.98 (0.73–1.31)
Diagnosed baseline	10,142	51	1.30 (1.04–1.61)	1.27 (0.95–1.69)
Hypothyroidism men				
No	78,193	543	1.00	1.00
Undetected	966	10	0.78 (0.46–1.30)	0.68 (0.36–1.28)
Diagnosed during follow-up	4081	33	1.00 (0.72–1.37)	1.09 (0.77–1.56)
Diagnosed baseline	1060	13	1.10 (0.67–1.80)	1.27 (0.73–2.21)
Type 2 diabetes mellitus				
No	205,330	830	1.00	1.00
Undetected	9272	97	1.62 (1.36–1.94)	1.60 (1.30–1.98)
Diagnosed during follow-up	20,018	173	1.33 (1.16–1.52)	1.29 (1.09–1.53)
Diagnosed baseline	6129	91	1.84 (1.55–2.19)	1.96 (1.57–2.44)
Hypertension				
No	292,257	594	1.00	1.00
Undetected	101,212	417	0.96 (0.86–1.06)	0.95 (0.84–1.08)
Diagnosed during follow-up	85,004	597	1.12 (1.01–1.24)	1.16 (1.03–1–30)
Diagnosed baseline	59,097	588	1.28 (1.16–1.42)	1.32 (1.17–1.49)

*HUNT* The HUNT Study, *HR* hazard ratio, *CI* confidence interval

^a^Participants with missing data in fully adjusted analyses excluded

^b^Model 1; adjusted for age. Model 2; Model 1 + sex, body mass index, smoking status, educational level, long term limiting illness, physical activity, serum cholesterol, and serum creatinine

### Type 2 diabetes mellitus

Both persons reporting and persons with undetected T2DM had increased mortality, compared to persons without T2DM; HR 1.69 (1.55–1.84) and HR 1.21 (1.08–1.37), respectively (Table 3). In persons attending both HUNT2 and HUNT3, mortality was increased regardless of disease status (undetected, diagnosed during follow-up, or diagnosed at baseline), compared with persons without the diseases (Table 4).

### Hypertension

Mortality was increased in persons reporting hypertension at HUNT2 (HR 1.23 (1.09–1.39)), however not in persons with undetected hypertension (HR 0.93 (0.82–1.05)), compared to normotensive persons (Table 3). Reclassification of hypertension status defining “recommended follow-up examination but not to start medicine” as normotensive if BP <140/90, or undetected hypertension if BP ≥140/90 gave us similar figures in the cox PH analyses as with the original classification: undetected hypertension; HR 0.95 (0.85–1.06), diagnosed hypertension; HR 1.23 (1.09–1.40).

In persons attending HUNT2 and HUNT3, mortality was increased among both persons diagnosed at baseline and during follow-up (Table 4). However, the HR was higher in persons with the longer disease history; HR 1.32 (1.17–1.49) and 1.16 (1.03–1.30) respectively.

## Discussion

Our data from the large, general population-based HUNT Study support previous evidence of increased mortality in persons with diagnosed T2DM and hypertension, compared to persons without the diseases. Undetected T2DM was also associated with increased mortality, however not undetected hypertension and hypothyroidism. In general, adjustments for selected covariates in the cox PH analyses did not alter the associations substantially.

### Hypothyroidism

The results regarding hypothyroidism must be interpreted cautiously owing to few deaths in this study-part. Previous studies of the association between hypothyroidism and mortality have varying designs and have presented conflicting results [17]. One Danish register-based study found increased mortality in persons with hypothyroidism, whilst a Scottish general population study did not [33, 34]. Neither of these included persons with undetected hypothyroidism, nor accounted for disease duration. Similar to our study, comorbidity were more frequent in persons with diagnosed hypothyroidism. We have not identified any studies analysing mortality in undetected hypothyroidism directly, but Laulund et al. found increased mortality with increased duration of elevated TSH, further; that mortality was increased in overt hypothyroidism opposed to subclinical hypothyroidism [21]. However, this association was only found among persons diagnosed in a hospital setting and that in addition had comorbidity.

According to the HUNT2 protocol, participants were recommended to consult their general practitioner (GP) if TSH > 5 mU/L (within 2-3 weeks if FT_4_ < 8 pmol/L, within 2 years if FT_4_ 8-20 pmol/L). Presumably, some persons with abnormal tests at the screening would have normal results at re-testing; hence we expect to have classified some persons without hypothyroidism as undetected hypothyroidism in our study. This should weaken any association with mortality in this group. Persons confirmed with hypothyroidism at re-testing would likely receive thyroxin supplement. Left untreated, severe hypothyroidism will ultimately lead to death, but even when treated there is some evidence for increased mortality [33]. Any misclassification of persons with undetected hypothyroidism in HUNT2, that became diagnosed after the survey, should however be accounted for in the supplementary analyses among those participating in both HUNT2 and HUNT3.

### Type 2 diabetes mellitus

Antidiabetic lifestyle changes and/or medical treatment in persons with diagnosed T2DM could decrease all-cause mortality, compared with undetected cases [35]. Nevertheless, in line with others, we found that the HR was higher in persons with diagnosed T2DM than in persons with undetected T2DM [36–38]. In the sensitivity analyses we found that duration of diagnosed T2DM seemed to increase the hazard, but also that persons with undetected T2DM between HUNT2 and HUNT3 had increased mortality.

Few studies have addressed mortality in undetected T2DM, but Figueiredo et al. found that undetected T2DM was associated with increased mortality after hospital admission with acute myocardial infarction [19]. However, on a population level, two systematic reviews did not find screening for T2DM to improve survival [14, 15].

### Hypertension

In line with previous knowledge [1], persons with diagnosed hypertension had increased mortality compared to normotensive persons. Somewhat unexpected however; persons with undetected hypertension did not have increased mortality, opposing the results of Barengo et al. [22]. This was neither found in the subset having undetected hypertension both at HUNT2 and HUNT3. In existing literature the relationship between undetected hypertension and mortality has barely been addressed. Although a systematic review presumed benefit on survival of screening for hypertension, there is no convincing evidence for it [4].

As with T2DM, confounding by duration of disease explain much of the difference. We found that persons diagnosed at HUNT2 had higher HR than persons diagnosed between the surveys. Previous cohort studies have found contradictory effects of antihypertensive treatment on all-cause mortality, [22, 39] however lack of control of disease duration is a common weakness. Randomized clinical trials have confirmed reduction in mortality by certain antihypertensive drugs in persons with diagnosed hypertension, [8] however not in mild hypertension [9].

The analyses of the reclassified hypertension status indicated that mortality in fact was increased also in participants reporting “recommended follow-up”, not only in the “start or continue taking medicine for high BP” group, hence the original categorization should be valid.

### Covariables

Serum cholesterol was not associated with all-cause mortality in any of the adjusted analyses. Petursson et al. neither found cholesterol to be associated with all-cause, nor cardiovascular mortality in the HUNT population [40]. The results are conflicting however, since Mørkedal el al. showed that total cholesterol to HDL cholesterol was associated with cardiovascular mortality [41]. The association obviously depend on the inclusion and exclusion criteria. Globally, total serum cholesterol is characterized as a risk factor for cardiovascular disease and included in risk calculators [42]. However, especially in elderly cholesterol’s relation with mortality is debated [43].

### Study limitations

In Norway, there is no national registry for these diseases; hence we relied on self-reported disease status in all classifications. Information bias, causing differential misclassification would occur if correct classification of self-reported hypothyroidism, T2DM, or hypertension was different in participants that survived and those who died. Persons with severe disease might be more reliable in reporting the disease compared to persons with mild disease, resulting in increased HR for mortality in the diagnosed groups. We expect misclassification of persons with the disease as not having the disease to attenuate any differences between the groups.

Misclassification owing to use of measurements taken at one time-point, and for the T2DM study part; use of non-fasting serum glucose, is a possibility in our study. We expect some participants with measurements outside the reference range would regress towards the mean if measured later. Such misclassification should weaken any relationship with mortality in these groups (under the assumption that persons without disease live longer).

During the three surveys of the HUNT Study (mid 80thies, mid 90thies, and 2006–08), it is possible that our study population has been exposed for some degree of screening effects; affecting external validity of our results. The resulting case finding might have influenced identification of disease at an early stage, such that the proportion with undetected disease could be lower than in other populations. This could underestimate any association with mortality among undetected cases. We have limited data whether participants were diagnosed between the HUNT survey on the basis of specific symptoms, opportunistic case finding, or other reasons. Such information could probably help to explain different mortality-rates. Further, the relatively high proportion of undetected hypertension could be a result of misclassification. Usually GPs do not give the diagnosis of hypertension before follow-up measurements or 24-h measurements confirm the diagnosis. This might weaken any association between undetected hypertension and mortality. During the review process we chose to perform an additional analysis to evaluate this potential misclassification. The diagnostic cut-off defining undetected hypertension in participants not reporting hypertension was now ≥160/100. However, the result was similar compared with the original analyses: Undetected hypertension was not associated with increased mortality (HR 1.06 (95%CI 0.93–1.20)), compared with persons without hypertension.

### Study strengths

The main strengths of the study were the population based cohort design, a high number of participants and the wide spectrum of diseases and characteristics. Similar results in the original and in the sensitivity analyses indicated that participant’s classification of own disease status was valid (no evidence for recall bias). Inclusion of analyses of disease duration is a further strength in this study.

## Conclusion

Participants with diagnosed T2DM or hypertension had increased mortality compared to participants without the diseases. Persons with undetected T2DM had increased mortality compared with persons without T2DM, but the HR was lower than among persons with diagnosed T2DM. Further, persons with undetected hypertension had life-expectancy comparable to persons without hypertension. Thus, the importance of screening for T2DM and hypertension on a general population level is still questionable. Future research should address in more detail the differences between undetected and diagnosed persons; as such knowledge can help clarify any potential benefit of active case finding or targeted screening in certain high-risk groups.

## Abbreviations

BMI: Body mass index
BP: Blood pressure
CI: Confidence interval
DM: Diabetes mellitus
FT_4_: Free thyroxin
GP: General practitioner
HbA1c: Hemoglobin A1c
HR: Hazard ratio
HUNT: Nord-Trøndelag Health Study
IQR: Inter quartile range
NTNU: Norwegian University of Science and Technology
PH: Proportional hazard
SD: Standard deviation
SRH: Self-rated health
T2DM: Type 2 diabetes mellitus
TSH: Thyroid stimulating hormone
WHO: World Health Organization
